# A pan‐genotype hepatitis C virus viral vector vaccine generates T cells and neutralizing antibodies in mice

**DOI:** 10.1002/hep.32470

**Published:** 2022-05-19

**Authors:** Timothy Donnison, Joey McGregor, Senthil Chinnakannan, Claire Hutchings, Rob J. Center, Pantelis Poumbourios, Paul Klenerman, Heidi E. Drummer, Eleanor Barnes

**Affiliations:** ^1^ Nuffield Department of Medicine Peter Medawar Building for Pathogen Research University of Oxford Oxford UK; ^2^ 534133 Burnet Institute Melbourne Victoria Australia; ^3^ Department of Microbiology and Immunology at The Peter Doherty Institute for Infection and Immunity University of Melbourne Parkville Victoria Australia; ^4^ 534133 Department of Microbiology Monash University Clayton Victoria Australia; ^5^ Nuffield Department of Medicine Jenner Institute University of Oxford Oxford UK

## Abstract

**Background and Aims:**

A prophylactic vaccine targeting multiple HCV genotypes (gt) is urgently required to meet World Health Organization elimination targets. Neutralizing antibodies (nAbs) and CD4^+^ and CD8^+^ T cells are associated with spontaneous clearance of HCV, and each may contribute to protective immunity. However, current vaccine candidates generate either nAbs or T cells targeting genetically variable epitopes and have failed to show efficacy in human trials. We have previously shown that a simian adenovirus vector (ChAdOx1) encoding conserved sequences across gt1‐6 (ChAd‐Gt1‐6), and separately gt‐1a E2 protein with variable regions deleted (E2Δ123_HMW_), generates pan‐genotypic T cells and nAbs, respectively. We now aim to develop a vaccine to generate both viral‐specific B‐ and T‐cell responses concurrently.

**Approach and Results:**

We show that vaccinating with ChAd‐Gt1‐6 and E2Δ123_HMW_ sequentially in mice generates T‐cell and antibody (Ab) responses comparable to either vaccine given alone. We encoded E2Δ123 in ChAdOx1 (ChAd‐E2Δ123) and show that this, given with an E2Δ123_HMW_ protein boost, induces greater CD81‐E2 inhibitory and HCV‐pseudoparticle nAb titers compared to the E2Δ123_HMW_ prime boost. We developed bivalent viral vector vaccines (ChAdOx1 and modified vaccinia Ankara [MVA]) encoding both Gt1‐6 and E2Δ123 immunogens (Gt1‐6‐E2Δ123) generating polyfunctional CD4^+^ and CD8^+^ T cells and nAb titers in prime/boost strategies. This approach generated nAb responses comparable to monovalent E2Δ123 ChAd/MVA vaccines and superior to three doses of recombinant E2Δ123_HMW_ protein, while also generating high‐magnitude T‐cell responses.

**Conclusions:**

These data are an important step forward for the development of a pan‐genotype HCV vaccine to elicit T cells and nAbs for future assessment in humans.

## Graphical abstract

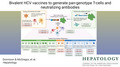

**Abbreviations**
Ab: antibody
Ag: antigen
BSA: bovine serum albumin
ChAd: chimpanzee adenovirus
ChAdOx1: chimpanzee adenovirus Oxford 1 (licensed vector)
E2: envelope 2 protein
GC: germinal center
Gt: genotype
HCVcc: HCV cell culture
HCVpp: HCV pseudoparticle
HMW: high molecular weight
ICS: intracellular cytokine staining
HVR: hypervariable region
ID_50_
: median infective dose
IFN: interferon
MVA: modified vaccinia Ankara
nAb: neutralizing antibody
NS: nonstructural protein
RBD: receptor binding domain

## INTRODUCTION

HCV currently infects >50 million persons and, despite the rollout of efficacious directly acting antiviral (DAA) drugs, remains endemic in many countries. The World Health Organization has set targets for HCV elimination by 2030. However, estimates suggest that most (>60%) countries will fail to meet these targets,^[^ ^1^, ^2^ ^]^ and therefore a vaccine will be essential for HCV elimination. In support of vaccine efforts, mathematical models of HCV transmission suggest that a prophylactic vaccine that was only 30% effective would significantly reduce HCV incidence used alongside diagnostics and DAA therapy.^[^ ^3^, ^4^, ^5^ ^]^ Given that liver disease caused by HCV requires chronic infection, the aim of a prophylactic vaccine should be to prevent chronic infection, rather than providing sterilizing immunity.

Spontaneous clearance occurs in 20% of primary HCV infections,^[^ ^6^ ^]^ and these persons are more likely to clear subsequent infections.^[^ ^7^ ^]^ Although the immune correlates that confer HCV resolution are not completely understood, data suggest that both T‐cell and antibody (Ab) responses play a crucial role in HCV clearance. These include the association of viral clearance with class I and II human leukocyte antigens,^[^ ^8^ ^]^ detection of broad polyfunctional T cells,^[^ ^9^ ^]^ and the early appearance of broadly neutralizing Abs.^[^ ^10^, ^11^ ^]^ Furthermore, T‐cell blocking studies in chimpanzees highlight the importance of a synergistic CD4^+^ and CD8^+^ T‐cell response.^[^ ^12^, ^13^ ^]^ The optimal HCV vaccine should generate protective humoral and cellular responses and be affordable and practical, both in terms of manufacture and delivery.

Current vaccine efforts have primarily focused on either inducing Abs targeting HCV surface envelope glycoproteins (gpE1E2^[^ ^14^, ^15^, ^16^, ^17^ ^]^) to block viral entry or activating T cells to destroy HCV‐infected cells.^[^ ^18^, ^19^, ^20^ ^]^ Vaccine development is challenged by HCV genetic diversity that facilitates viral escape from Abs and T cells. Only two vaccines have advanced to human trials,^[^ ^15^, ^16^, ^17^, ^18^, ^19^, ^20^ ^]^ and both have elicited immune responses to antigens (Ags) that display high sequence diversity and are prone to mutational escape (reviewed by Bailey et al.^[^ ^21^ ^]^). One of these used a chimpanzee adenovirus (ChAd) vector (*ChAd3*) and modified vaccine Ankara (MVA) vector prime/boost strategy, encoding an HCV gt‐1b nonstructural (NS) sequence generating high‐magnitude, polyfunctional CD4^+^ and CD8^+^ T cells.^[^ ^20^ ^]^ These T cells predominantly targeted gt‐1b epitopes that exhibited high sequence variability with limited reactivity to non‐1b genotypes (gts)^[^ ^20^, ^22^ ^]^ and failed to prevent chronic HCV in a phase II clinical trial.^[^ ^23^ ^]^ The other vaccine consists of recombinant full‐length gt‐1a gpE1E2 protein. Despite the generation of high‐Ab titers, neutralizing antibodies (nAbs) against heterologous HCV pseudoparticles (HCVpp) were generated in a minority of healthy volunteers in a phase I human trial.^[^ ^15^, ^16^, ^17^ ^]^ These data show that alternative vaccine strategies that target multiple HCV gts, generating both nAbs and CD4^+^/CD8^+^ T cells, are urgently required to maximize the chances of vaccine efficacy.

We have previously described the development of a chimpanzee adenovirus Oxford 1 (ChAdOx1) vector encoding conserved HCV sequences across gt‐1 to ‐6 (ChAd‐Gt1‐6) that generates robust T‐cell responses targeting conserved epitopes in mice.^[^ ^24^, ^25^ ^]^ We also generated an Ab vaccine using a modified HCV gpE2 vaccine candidate with deletions of hypervariable domains, hypervariable region (HVR) 1, HVR2, and intergenotypic variable region (IgVR) presented as a disulfide‐linked, high‐molecular‐weight (HMW) antigen (E2Δ123_HMW_). When used to immunize guinea pigs, E2Δ123_HMW_ increased sera‐neutralizing activity against heterologous HCV strains by targeting conserved epitopes on the neutralizing face of the envelope 2 protein (E2) receptor binding domain (RBD).^[^ ^26^ ^]^ Here, we describe vaccine strategies to combine these two HCV vaccines to generate immune responses to conserved epitopes, including sequential vaccinations and heterologous prime‐boost using both protein, ChAd, and MVA vectors to determine the optimal strategy for generating cross‐genotype T‐ and B‐cell responses.

## MATERIALS AND METHODS

### Viral vector vaccine nomenclature

The ChAdOx1 and MVA vector designs (Figure S1) encode either: (1) the conserved HCV sequence segment of gt‐1 to ‐6 (Gt1‐6)^[^ ^25^ ^]^; (2) the E2Δ123 sequence; or (3) a bivalent immunogen encoding the Gt1‐6 and E2Δ123 sequences.

### Protein vaccine nomenclature

The recombinant protein, E2Δ123, encodes E2_410‐661_ with deleted HVR1 (E2_384‐409_), HVR2 (E2_460‐485_), and IgVR (E2_570‐580_) sequences (Figure S2A). The HMW E2Δ123 species is called E2Δ123_HMW_, and the monomer is called E2Δ123. The recombinant monomeric protein, E2RBD, is wild type (WT) and encodes E2_384‐661_, the full length of the E2 receptor binding domain. Both E2Δ123_HMW_ and E2RBD proteins have a C‐terminal polyhistidine tag for purification and application in assays. The E2Δ123 and E2RBD sequences were designed from Genbank sequence AF 009606.

### Vaccine production

A human codon optimized HCV immunogen was inserted into the donor vector between the cytomegalovirus promoter and bovine growth hormone poly‐A sequence by In‐Fusion cloning to generate the shuttle vector. The shuttle vector was cloned into the ChAdOx1 destination vector, using ThermoFisher’s LR gateway cloning method, and linearized using *PmeI* restriction and transfected into T‐REx‐293 cells (ThermoFisherScientific) to generate ChAdOx1 vaccines. For MVA vaccines, the HCV immunogen was cloned at the F11 locus of the pMVA‐shuttle vector and the recombinant MVA generated by recombining the pMVA‐shuttle vector and WT MVA. HCV viral vector vaccines were manufactured by the Viral Vector Core Facility (Jenner Institute, University of Oxford, Oxford, UK).

Recombinant proteins were expressed using the Freestyle 293‐F cells stable transfected cell clone, purified from the culture supernatants using cobalt‐charged TALON metal affinity resin (Clontech) by the C‐terminal polyhistidine tag followed by size‐exclusion chromatography on a Superdex200 prep grade 16/600 column (GE Healthcare), using the ÄKTA pure fast protein liquid chromatography system (GE Healthcare) equilibrated in PBS (pH 6.8). Fractions containing the HMW species were pooled and concentrated through an Amicon® 30‐kDa molecular‐weight cut‐off ultracentrifugal device (Merck).

### Animal experiments

Mouse studies were performed at the Biomedical Services Building (BSB), Oxford, according to UK Home Office Regulations (project license numbers P874AC0F0 and PP3430109) and approved by the ethical review board at the University of Oxford. All experiments complied with the ARRIVE (Animal Research: Reporting of In Vivo Experiments) guidelines and UK Animals (Scientific Procedure) Act, 1986. Groups of 4‐12 age‐matched 6‐ to 8‐week‐old female *C57BL/6* mice were used throughout and housed at a pathogen‐free facility in individually vented cages and fed a nutrient diet (Harlan Teklad Lab Blocks). Mice were vaccinated 1 week after arrival (i.m., 26‐G needle; during light cycle; nonblinded) and culled postvaccination by schedule‐1 (CO_2_ exposure followed by cervical dislocation or heartbeat cessation).

### Organ isolation and processing

Terminal bleeds (cardiac puncture) were refrigerated at 4°C and sera isolated within 24 h. Spleens were mechanically processed using a sterile plunger and a 40‐μm cell strainer. Splenic red blood cells were lysed with ACK (Ammonium‐Chloride‐Potassium) Lysis Buffer (ThermoFisherScientific) for <1 min and remaining cells resuspended in R10 media (RPMI‐1640 media with l‐glutamine [5%], penicillin‐streptomycin [5%], and 10% fetal calf serum). Cell yields were calculated using a Guava Personal Cell analysis system (Merck Millipore 0100‐14230) and the Muse Cell Analyser (Merck Millipore).

### Peptides and Antibodies

HCV genotype‐1a (H77; Genbank NC_038882), ‐1b (J4; Genbank AF054250), and ‐3a (K3a650; Genbank D28917) peptides of 15‐18 amino acids, overlapping by 11 amino acids spanning the whole HCV proteome (optimal for CD4^+^ and CD8^+^ T‐cell activation) were obtained from the National Institutes of Health (Bethesda, MD) and used in T‐cell assays. Each peptide was initially dissolved in DMSO (40 mg/ml) and combined into nine peptide pools (300 μg/ml) covering the sequences of: Core, E1, E2, NS3p, NS3h, NS4, NS5a, NS5bI (aa2421‐2718 in H77 reference sequence), and NS5bII (aa2719‐ 3011). Synthetic peptides for ELISAs were synthesized (Genscript) and contained an N‐terminal biotin group for avidin capture (Table S1). Antibodies used are described in Table S2.

### *Ex vivo* IFNγ Enzyme‐Linked Immunospot

*Ex vivo* detection of interferon (IFN)γ by HCV‐stimulated splenocytes with enzyme‐linked immunospot assays were performed as described.^[^ ^24^ ^]^ Total and peptide‐pool–specific (IFNγ^+^ spot forming units) responses were background subtracted and plotted per 10^6^ splenocytes.

### Intracellular cytokine staining

Intracellular cytokine staining (ICS) was performed as described.^[^ ^24^ ^]^ Gating/analysis were performed in FlowJo (v10.7; TreeStar, Ashland, OR). Cytokine coexpression within T‐cell subsets was determined using Boolean gating in FlowJo and graphs produced in Pestle (v2.0), and SPICE (v6.0; NIAID, NIH).

### ELISA

Direct‐binding assays and synthetic peptide ELISAs were performed as described.^[^ ^27^ ^]^ Antibody titers were calculated as the reciprocal dilution of serum required for a 10‐fold increase over binding to bovine serum albumin.

### Antibody isotype assay

Antibody isotype assays were performed as described for ELISAs, with the addition of goat antimouse ‐IgG1, ‐IG2a, ‐IG2b, ‐IG3, ‐IgA, and ‐IgM added at a 1:1000 dilution in 0.5% BSA in PBS containing 0.05% Tween 20 (BSA5PBST; 1 mg/ml)/well (Mouse Monoclonal Ab Isotyping Reagent Kit; Sigma‐Aldrich) after addition of the serum. Bound Abs were detected with antigoat horseradish peroxidase (HRP) (Invitogen). Positive controls were mAb44 (IgG1 control), mAb25 (IgG2a control), mAb24 (IgG2b control), and mAb64 (IgG3) anti‐HCV Abs at 1 μg/ml in BSA5PBST.

### CD81‐LEL inhibition assay

The ability of immune serum to prevent the interaction between E2 and recombinant CD81‐LEL has been described,^[^ ^27^ ^]^ with the exception that bound E2 was detected by using humanized mouse mAb2A12 followed by polyclonal goat antihuman HRP‐conjugated Ab (Dako). Data was analyzed using GraphPad Prism software (v9.0; GraphPad Software Inc., La Jolla, CA) where inhibitory titers were expressed as the reciprocal dilution of immune serum that reduces the binding reaction being competed by 50% (median infective dose [ID_50_]) where binding in the absence of sera is equivalent to 100% binding.

### HCVpp and cell‐culture neutralization assay

HCV neutralization assays were performed as described.^[^ ^28^ ^]^ Briefly, HEK293T cells were cotransfected in a 1:1 (w/w) ratio of pE1E2H77c (gt‐1a; Genbank AF011751) or pUKN3A1.9 (gt‐3a; Genbank AY734985) and pNL4–3.LUC.R‐E‐ to produce HCVpp. Alternatively, infectious HCV cell culture (HCVcc)‐derived genotype 3a (S52; Genbank EU204645), and 5a (SA13; Genbank FJ393024) were produced by transfecting Huh7.5 cells with *in vitro*–transcribed RNA by electroporation,^[^ ^26^ ^]^ with the modification that luciferase activity was measured in a microplate reader (ClarioSTAR; BMG Labtechnologies). Mean percentage entry was calculated as (relative light units [RLU] plasma + virus)/(RLU medium+virus) × 100. Percentage entry was plotted against the reciprocal dilution of plasma in GraphPad Prism software (version 9; GraphPad Software) and curves fitted with a one‐site specific binding Hill plot. The reciprocal dilution of plasma required to prevent 50% virus entry was calculated from the nonlinear regression line (ID_50_). The lowest amount of nAb detectable was a titer of 40. Samples that did not reach 50% neutralization were assigned an arbitrary value of 20.

### Statistical analysis

Data were analyzed using GraphPad Prism (v9.0; GraphPad Software). Nonparametric Mann‐Whitney U or Kruskal‐Wallis tests with multiple comparisons were used to determine significant differences between group medians at 95% CIs. *p* values <0.05 indicate a significant difference: *p < 0.05; **p < 0.01; ***p < 0.001; ****p < 0.0001.

## RESULTS

### ChAd‐E2Δ123 prime increases CD81 binding inhibition and nAb titers

**FIGURE 1:**
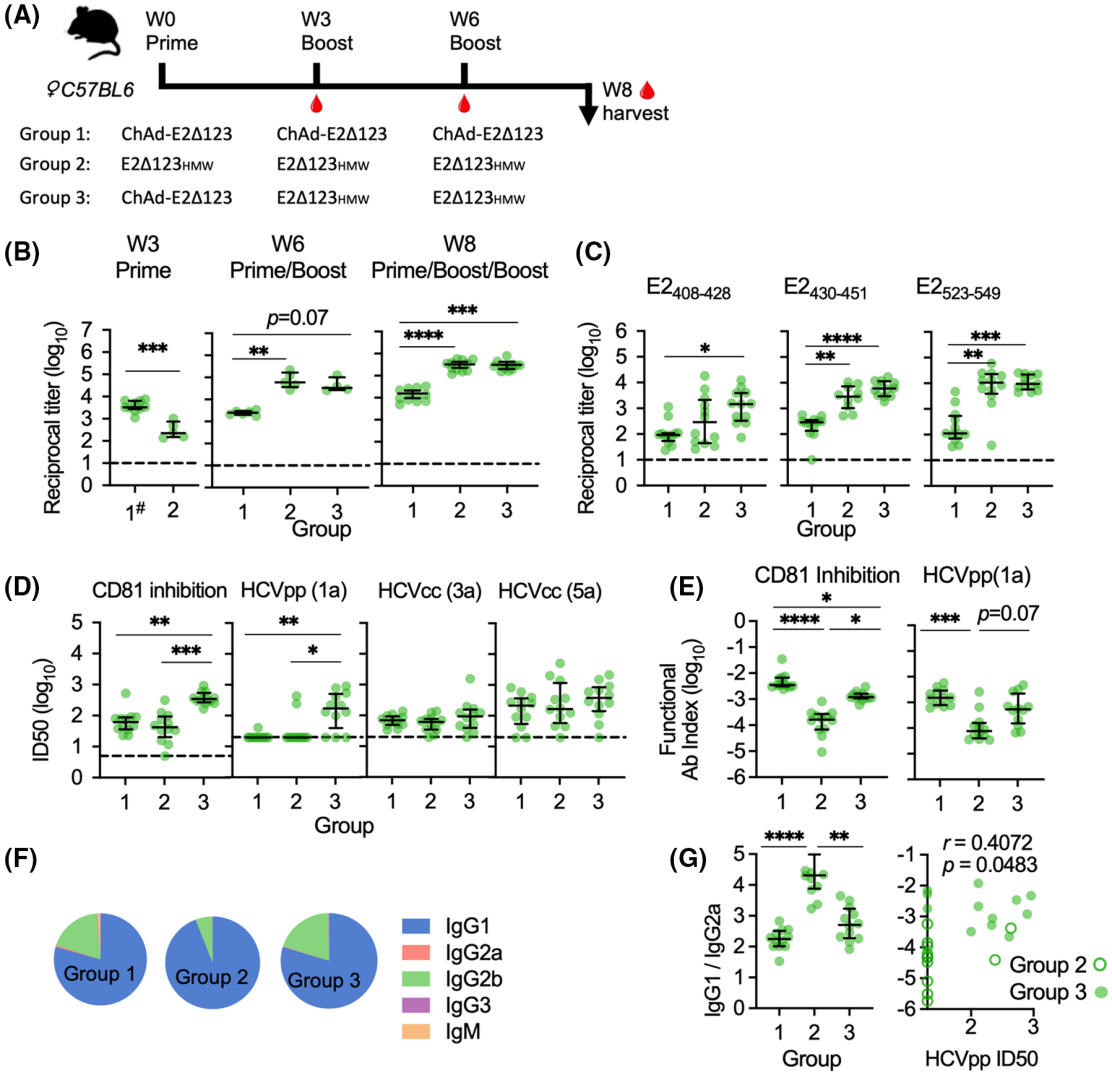
Immunogenicity of an E2Δ123 protein sequence encoded in a ChAdOx1 prime vaccine. (A) Groups of 12 age‐matched female C57BL/6 mice were vaccinated with a prime vaccine at day 0, a boost vaccine at week 3 (W3), and another boost vaccine at week 6 (W6), followed by a terminal bleed at week 8 (W8). Group 1 had three sequential ChAd‐E2Δ123 vaccinations; group 2: three sequential E2Δ123_HMW_ protein vaccinations; and group 3, a ChAd‐E2Δ123 prime followed by two sequential E2Δ123_HMW_ protein boosts. ChAd‐E2Δ123 was given i.m. in the left quadricep at 10^8^ infectious units (IU) in 40 μL sterile PBS. E2Δ123_HMW_ (20 μg) was mixed 1:1 with 50 μL of Addavax® adjuvant and administered s.c. in the scruff ofthe neck. (B) Sera was collected at week 3 (preboost 1; hash indicates pooled groups 1 and 3), week 6 (preboost 2), and at week 8 (end of study; EOS) to determine the Ab titers’ capacity to bind gt‐1a E2Δ123 monomer in an ELISA. (C) Immune sera’s (W8, EOS) capacity to bind CD81 binding determinants: AS412 (E2_408‐428_); AS434 (E2_430‐451_); and CD81 binding loop (E2_523‐549_). ELISA Ab titers were measured at 10 times background (BSA), using dilution curves, and are plotted as log reciprocal titers. (D) EOS immune sera titers that inhibit 50% (ID_50_) of: E2 binding to CD81; neutralizing HCV gt‐1a HCVpp; and HCV gt‐3a and ‐5a HCVcc virion entry into Huh7.5 cells. ID_50_ inhibitory titers were determined using a dilution curve where the vaccine‐induced Ab response was background subtracted and standardized to a negative control, which displayed 100% binding/entry (e.g., BSA, instead of immune sera, incubated with E2 or HCVpp/cc before determining CD81 binding or cell infectivity). (E) ID_50_ inhibitory titer of immune sera as a function of the overall Ab titer, displayed as Ab titer divided by ID_50_ titer. (F) EOS E2‐specific Ab isotype titers are displayed as a fraction of the total Ab titer (median pie base). (G) IgG2a titer of immune sera was calculated as a function of the IgG1 titer and is displayed as IgG1 titer divided by IgG2a titer (IgG1/IgG2a). The lower the titer, the closer the IgG2a to IgG1 ratio is to 1:1, indicative of IgG1 to IgG2a class switching. The reciprocal titer (1/IgG1:IgG2a) was plotted against the HCVpp ID_50_ titer for immune sera from animals that received C/P/P and P/P/P to determine any correlation. All bars are medians, and interquartile ranges are displayed. For Panel B–D, the dashed line is the cutoff for detectable responses. The D’Agostino and Pearson test was used to determine normality of data distribution, and Mann‐Whitney U tests were performed to determine significant differences between two group medians at a 95% CI (Kruskal‐Wallis test for multiple groups). *p* values indicate a significant difference between groups when: *p < 0.05; **p < 0.01; ***p < 0.001; ****p < 0.0001

Sequential administration of ChAd‐Gt1‐6 and E2Δ123_HMW_ protein did not alter the immune response to either vaccine (Figures S1–S3). Therefore, we developed viral vectors that encode the E2Δ123 sequence alongside T‐cell immunogens, to develop a single‐viral–vectored vaccine to prime both T‐ and B‐cell responses. First, we generated a ChAdOx1 vector encoding the E2Δ123 sequence (Figure S1B) and measured Ab titers specific for E2Δ123 and neutralization epitopes, Ab neutralizing capacity, and Ab isotype profile and compared these to responses generated with E2Δ123_HMW_ protein vaccine. We used homologous (three ChAd‐E2Δ123 or E2Δ123_HMW_ protein vaccines only; groups 1 and 2) and heterologous (ChAd‐E2Δ123 prime with two E2Δ123_HMW_ protein boosts; group 3) prime‐boost‐boost regimens (Figure 1A). The ChAd‐E2Δ123 vaccine induced Ab titers assessed at 3 weeks postprime that were significantly higher than with the E2Δ123_HMW_ protein vaccine (***p = 0.0007; Figure 1B). However, after subsequent booster vaccinations at week 3 and week 6, homologous E2Δ123_HMW_ immune sera displayed greater Ab titers than homologous ChAd‐E2Δ123 immune sera (**p = 0.0065 [week 6] and ****p < 0.0001 [week 8] for group 1 vs. group 2; Figure 1B). Groups boosted with E2Δ123_HMW_ protein (groups 2 and 3) induced significantly higher week 8 Ab titers compared to their week 3 preboost titers, whereas the homologous ChAd‐E2Δ123 regimen (group 1) induced significantly higher week 8 Ab titers after a prime and boost (Figure S4A). Mice immunized with E2Δ123_HMW_ protein vaccine (groups 2 and 3) displayed significantly greater Ab titers to AS412 (E2_408‐428_), AS434 (E2_430‐451_), and the CD81 binding loop (E2_523‐549_) compared to mice immunized with ChAd‐E2Δ123 alone (Figure 1C). Strikingly, mice that received a ChAd‐E2Δ123 prime vaccination followed by two E2Δ123_HMW_ boost vaccinations (group 3) displayed greater inhibitory and gt‐1a neutralization titers compared to the homologous regimens (E2‐CD81 ID_50_: **p = 0.0016 for group 1 vs. group 3 and ***p = 0.0001 for group 2 vs. group 3; HCVpp ID_50_, **p = 0.0013 for group 1 vs. group 3 and *p = 0.0113 for group 2 vs. group 3, Figure 1D; 80% inhibitory dose in Figure S4B). When ID_50_ titers (CD81 inhibition and HCVpp) were assessed in relation to the overall Ab titer (functional Ab index: ID_50_/Ab titer), the ChAd‐E2Δ123 immune sera (groups 1 and 3) had a significantly higher functional index compared to E2Δ123_HMW_ immune sera, indicating a qualitative enhancement of the vaccine‐induced Ab response (Figure 1E). When analyzing the Ab response by isotype, regimens with an E2Δ123_HMW_ protein vaccine boost generated higher titers across all isotypes compared to the homologous ChAd‐E2Δ123 regimen (Figure S4C), in line with the overall Ab titers. However, the ChAd‐E2Δ123 prime, E2Δ123_HMW_ protein boost (group 3) regimen generated a higher IgG2a titer (*p = 0.0103 for group 2 vs. group 3; Figure S4C) and, overall, a broader isotype repertoire (Figure 1F). When the IgG2a titer was expressed as a function of the IgG1 titer (IgG1/IgG2a), ChAd‐E2Δ123 immune sera (groups 1 and 3) had a significantly lower reciprocal titer compared to E2Δ123_HMW_ immune sera, indicative of increased germinal center (GC) processing (****p < 0.0001 for group 1 vs. group 2; **p = 0.0032 for group 2 vs. group 3; Figure 1G). Finally, for E2Δ123_HMW_ immune sera (groups 2 and 3 combined), the IgG1:IgG2a reciprocal titer positively correlated with HCVpp‐neutralizing titers (*r* = 0.4072; *p* = 0.0483; Figure 1G). Overall, encoding E2Δ123 in ChAdOx1 enhanced the quality of the Ab response and, when combined with E2Δ123_HMW_ protein boosts, generated high functional Ab titers.

### Bivalent vaccines are as immunogenic as monovalent vaccines

The ability to generate a robust Ab response to E2Δ123 using viral vectors suggests that it is possible to engineer a viral vector to deliver both HCV‐specific T‐ and B‐cell Ags. Therefore, we designed ChAdOx1 and MVA vector vaccines that encode both the Gt1‐6 and E2Δ123 HCV sequences in a single immunogen (Gt1‐6‐E2Δ123; Figure S1C,F) to generate nAbs and T cells targeting multiple HCV gts.

**FIGURE 2:**
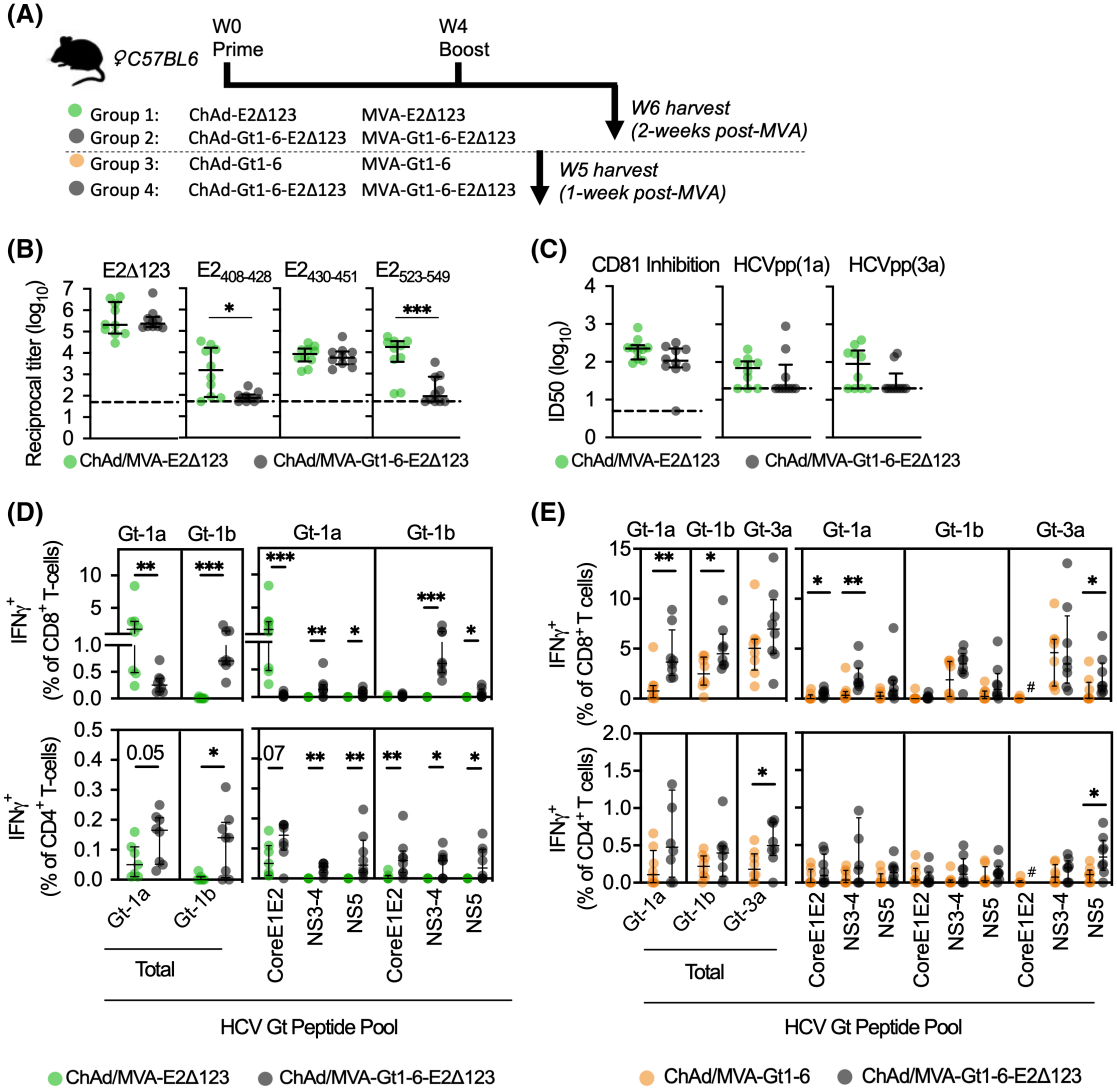
Immunogenicity of a bivalent HCV vaccine immunogen (Gt1‐6‐E2Δ123) compared to monovalent immunogens, Gt1‐6 and E2Δ123, in a ChAdOx1 prime, MVA boost regimen. Two experiments—each consisting of two groups of age‐matched female C57BL/6 mice (n = 10)—were conducted. (A) The first experiment consisted of a week 0 ChAd‐E2Δ123 prime (10^8^ infectious units [IU] in 40 μL of sterile PBS) followed by a week 4 MVA‐E2Δ123 (10^7^ plaque‐forming units [PFU] in 40 μL of sterile PBS group 1, green dots) versus a week 0 ChAd‐Gt1‐6‐E2Δ123 prime followed by a week 4 MVA‐Gt1‐6‐E2Δ123 (group 2, gray dots), all with Abs and splenic T cells analyzed at week 6 (week 2 post‐MVA). The second experiment compared the peak T‐cell response (week 1 post‐MVA, i.e., week 5 post‐ChAd) of a ChAd‐Gt1‐6 prime followed by an MVA‐Gt1‐6 (group 3, orange dots) versus a week 0 ChAd‐Gt1‐6‐E2Δ123 prime followed by a week 4 MVA‐Gt1‐6‐E2Δ123 (group 4, gray dots). In both experiments, ChAd and MVA vaccines were delivered via i.m. at 10^8^ infectious units and 10^7^ plaque‐forming units, respectively, in the left quadricep. (B) Group 1 and 2 immune sera were assessed for reactivity to E2Δ123 monomer and AS412 (E2_408‐428_), AS434 (E2_430‐451_), CD81 binding loop (E2_523‐549_), and (C) capacity to inhibit 50% of the binding of CD81 to E2 binding and neutralization of gt‐1a HCVpp virion entry into Huh7.5 cells. ID_50_ inhibitory titers were determined using a dilution curve where the vaccine‐induced Ab response was background subtracted and standardized to a negative control, which displayed 100% binding/entry (e.g., BSA, instead of immune sera, incubated with E2 or HCVpp before determining CD81 binding or cell infectivity). (D,E) Group 1 and 2 splenocytes were harvested at week 6 (D) and group 3 and 4 splenocytes at week 5 (E) and all stimulated *ex vivo* using HCV peptides (15 mer overlapping by 11 aa) covering the length of the HCV proteome (total) and for peptide pools: Core‐E1‐E2, NS3‐4, and NS5, for gt‐1a (H77), ‐1b (J4), or ‐3a (k3a650). IFNγ^+^ CD8^+^ and CD4^+^ T‐cell frequencies as a percentage of total CD8^+^ or CD4^+^ T‐cell frequencies were determined by intracellular cytokine staining and flow cytometry. All data are plotted as medians and interquartile ranges. For panel B and C, the dashed line is the cutoff for detectable responses. The D’Agostino and Pearson test was used to determine the normality of data distribution, and Mann‐Whitney U tests were performed to determine significant differences between two group medians at a 95% CI. *p* values indicate a significant difference between groups when: *p < 0.05; **p < 0.01; ***p < 0.001; ****p < 0.0001. aa, amino acids

In mice, a week 0 ChAd prime and week 4 MVA boost vaccine (the regimen referred to as “prime/boost” hereon in and shown as a schematic in Figure 2A), encoding either a (1) monovalent E2Δ123 immunogen (design in Figure S1B,E) or (2) bivalent Gt‐1‐6‐E2Δ123 immunogen (design in Figure S1C,F), was administered. Both regimens generated similar titers of Abs binding E2Δ123 and the AS434 (E2_430‐451_) neutralization epitope (Figure 2B), inhibiting CD81‐E2 binding and neutralizing both homologous and heterologous HCVpp (gt‐1a and ‐3a, ID_50_) at 2 weeks post‐MVA vaccination (Figure 2C; ID_80_ in Figure S5A). However, monovalent vaccine immune sera displayed greater titers of Abs targeting neutralization epitopes AS412 (E2_408‐428_) and the CD81 binding loop (E2_523‐549_; Figure 2B), compared to animals that received the bivalent vaccine. Furthermore, more animals within the prime/boost monovalent vaccine group generated nAb activity compared to those in the prime/boost bivalent vaccine group (7 of 10 vs. 3 of 10, respectively, for gt‐1a; 6 of 10 vs. 2 of 10, respectively, for gt‐3a; Figure 2C).

When assessing T‐cell responses, the prime/boost monovalent vaccine group generated a significantly higher E2‐specific CD8^+^ T‐cell response (gt‐1a, but not gt‐1b) compared to the prime/boost bivalent vaccine group (***p = 0.0003; Figure 2D). Conversely, the prime/boost bivalent vaccine group generated greater CD4^+^ and CD8^+^ T cells specific for nonstructural proteins (NS) 3‐4 and NS5 peptide pools (both gt‐1a and ‐1b peptide variants) than the prime/boost monovalent vaccine group (Figure 2D; core/E1‐specific responses were undetectable: Figure S5B). All CD8^+^ T cells displayed similar polyfunctionality, whereas monovalent vaccine‐induced CD4^+^ T cells had increased polyfunctionality (Figure S5D). When comparing the prime/boost bivalent vaccine regimen (group 4) against the prime/boost monovalent T‐cell vaccine regimen (group 3: ChAd‐Gt1‐6/MVA‐Gt1‐6), the prime/boost bivalent vaccine group induced greater T‐cell responses to NS3‐4 (gt‐1a; CD8^+^ T cells) and NS5 (gt‐3a; CD4^+^ and CD8^+^ T cells) and, overall, a significantly higher total of gt‐1a‐ and gt‐1b‐specific IFNγ^+^ CD8^+^ T‐cell (gt‐1a, **p = 0.0047; gt‐1b, *p = 0.0379; Figure 2E) and gt‐3a IFNγ^+^ CD4^+^ T‐cell responses compared to the prime/boost monovalent vaccine group (*p = 0.041; Figure 2E). All T cells exhibited polyfunctionality (Figure S5E). The gating strategy for all ICS data is shown (Figure S5F) with representative plots given (Figure S5G). These data suggest that a bivalent vaccination approach to induce HCV‐specific T cells may be preferable to using a monovalent T‐cell vaccine alone.

### A bivalent ChAd prime, MVA boost generates higher‐magnitude T cells and Ab titers than three E2Δ123_HMW_ protein vaccines

**FIGURE 3:**
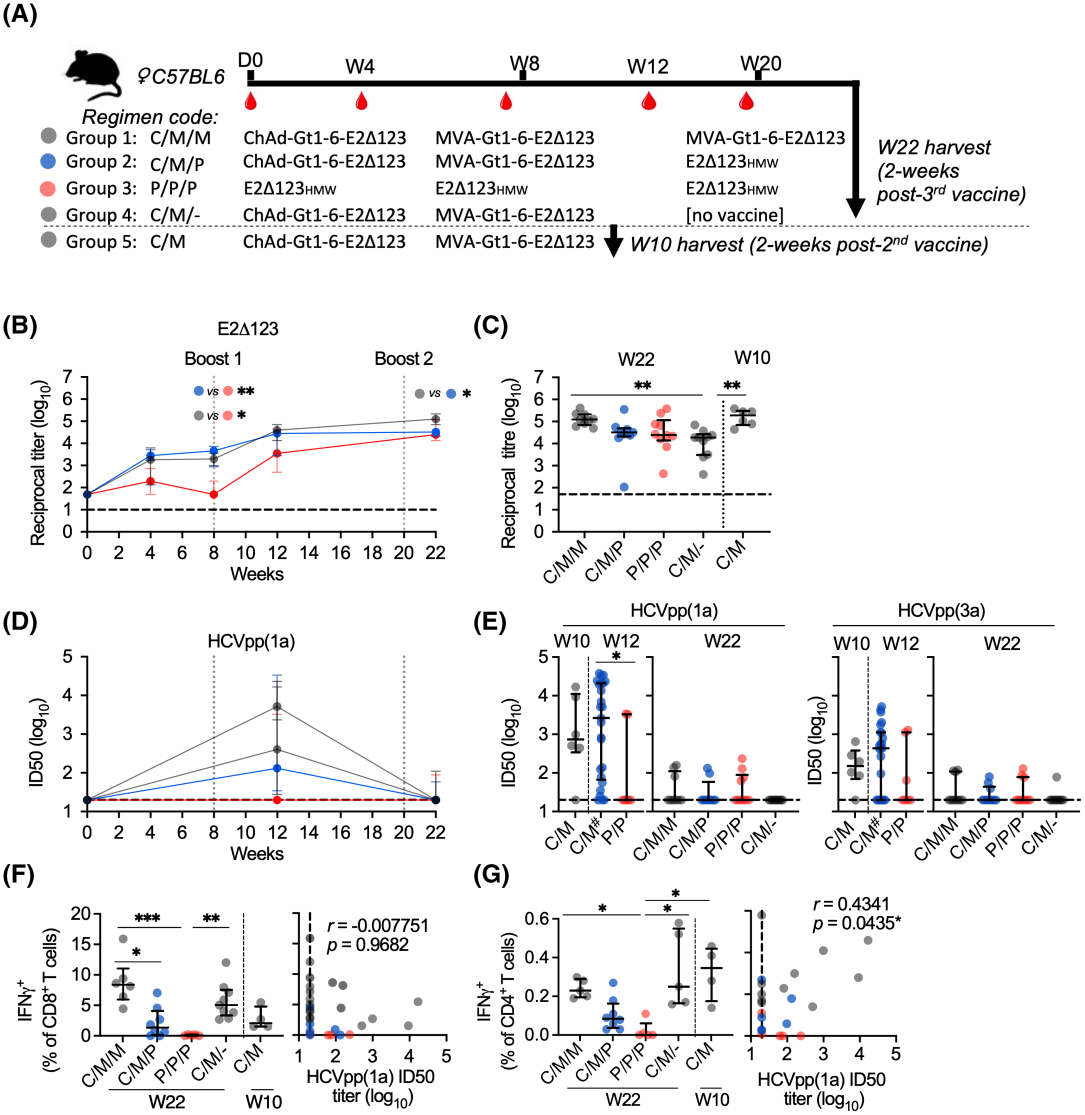
Assessment of mixed‐modality vaccination regimens using bivalent HCV vaccines and E2Δ123_HMW_ protein vaccines. (A) Groups of 10 age‐matched female C57BL/6 mice were vaccinated with a prime vaccine at day 0, a boost vaccine at week 8 (W8), and another boost vaccine at week 20 (W20), followed by a terminal bleed at week 22 (W22), 2 weeks after the last vaccination. ChAd‐Gt1‐6‐E2Δ123 was delivered via i.m. in the left quadricep at 10^8^ infectious units in 40 μL of sterile PBS, MVA was delivered via i.m. in the left quadricep at 10^7^ plaque‐forming units in 40 μL of sterile PBS, and E2Δ123_HMW_ (20 μg) was mixed 1:1 with 50 μL of AddaVax™ adjuvant and administered s.c. in the scruff of the neck. (B) Longitudinal ELISA assay analysis of immune sera collected at weeks 4, 8, 12, and 22 and plotted as Ab titer (log_10_) specific for the E2Δ123 monomer. (C) Comparison of week 10 and 22 ELISA assays between groups. (D) Longitudinal HCVpp (gt‐1a) assay analysis of immune sera collected at weeks 12 and 22 and plotted as ID_50_ (log_10_; dashed line is the cutoff for detectable responses). (E) Comparison of week 10, 12, and 22 HCVpp (gt‐1a and ‐3a) ID_50_ titers between groups. Hash indicates groups that were pooled together because of having the same vaccinations up until that point. (F,G) Splenocytes were harvested at week 22 and stimulated *ex vivo* using HCV peptides (15 mer overlapping by 11 aa) covering the length of the HCV proteome for gt‐1a (H77) and assessed by intracellular cytokine staining and flow cytometry to detect IFNγ^+^ CD8^+^ (F) and CD4^+^ (G) T cells and their correlation with their respective Ab titer in each animal. ICS data are plotted as a percentage of the parent group (live CD3^+^ CD4/8^+^). All data are plotted as medians and interquartile ranges. The D’Agostino and Pearson test was used to determine normality of data distribution, and Mann‐Whitney U tests were performed to determine significant differences between two group medians at a 95% CI (Kruskal‐Wallis test for multiple groups). *p* values indicate significant difference between groups when: *p < 0.05; **p < 0.01; ***p < 0.001; ****p < 0.0001. aa, amino acids

We next assessed mixed‐modality regimens using bivalent viral vector and E2Δ123_HMW_ protein vaccines in a week 0 prime (W0), week 8 boost (W8), and week 20 (W20) boost vaccination regimen (prime/boost/boost). For simplicity, vaccines are denoted “C” (ChAd‐Gt1‐6‐E2Δ123), “M” (MVA‐Gt1‐6‐E2Δ123), and “P” (E2Δ123_HMW_ protein; (Figure 3A). C/M/M and C/M/P vaccine immune sera displayed greater anti‐E2Δ123 Ab titers at week 8 (preboost 1) compared to P/P/P vaccine immune sera (Figure 3B; *p = 0.0247 and **p = 0.0037, respectively). At week 22, C/M/M vaccine immune sera displayed significantly greater anti‐E2Δ123 Ab titers compared to C/M/P vaccine immune sera (Figure 3B; *p = 0.038). Furthermore, week 22 C/M/M immune sera displayed significantly higher Ab titers targeting neutralization epitopes AS412 (E2_408‐428_) and AS434 (E2_430‐451_) compared to C/M/P and P/P/P (Figure S6A). No difference was observed between C/M (week 10) and C/M/M (week 22) Ab titers, but both regimens generated higher Ab titers than C/M/‐ (week 22), suggesting an Ab titer decline after the initial boost (Figure 3C).

Greater nAb titers against HCVpp(1a) were observed at week 12 after the first boost compared to week 22 after the second boost (Figure 3D; and Figure S6B). Only the P/P/P regimen generated higher nAb titers at week 22 compared to week 12, although these titers were low and not significantly different between time points (Figure S6B). Strikingly, week 12 nAb titers were greater in mice that received the ChAd‐Gt1‐6‐E2Δ123/MVA‐Gt1‐6‐E2Δ123 vaccines compared to mice that received two E2Δ123_HMW_ protein vaccines (gt‐1a, *p = 0.0365; Figure 3E), with no differences detected between regimens at week 22. This trend was also observed in CD81‐E2 inhibitory titers, although it was not statistically significant (Figure S6C).

Last, the C/M/M regimen generated greater IFNγ^+^ T‐cell frequencies compared to C/M/P and P/P/P regimens (CD8: Figure 3F, *p = 0.0307 and ***p = 0.0006; CD4: Figure 3G, *p = 0.0297). C/M/M‐induced CD8^+^ T cells were predominantly polyfunctional (IFNγ^+^ and TNFα^+^) in contrast to C/M/P CD8^+^ T cells (monofunctional), whereas CD4^+^ T cells from both regimens displayed similar functionality profiles (Figure S6D). Whereas no significant difference in T‐cell frequencies was observed between C/M and C/M/M regimens, these regimens generated significantly greater IFNγ^+^ CD4^+^ T‐cell responses compared to the P/P/P regimen (*p = 0.0175), which positively correlated with nAb titers (*r* = 0.4341; *p = 0.0435; Figure 3G). Overall, a single bivalent MVA boost vaccine generated the highest nAb titers while inducing a robust polyfunctional CD4^+^ and CD8^+^ T‐cell response, in contrast to a third vaccination (MVA or E2Δ123_HMW_ protein) or three sequential E2Δ123_HMW_ protein vaccinations.

**FIGURE 4:**
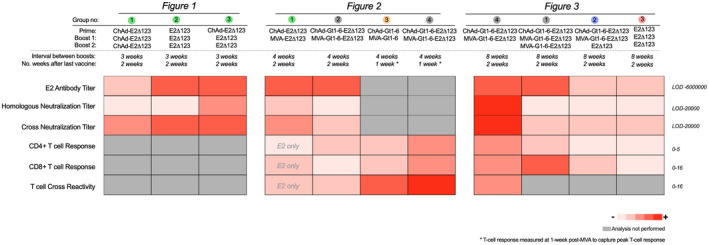
Summary of vaccination regimens. Comparison of 11 different vaccination regimens based on their immunogenicity, shown as a summary of presented data in Figures 1, 2, 3. Heatmap was generated using the median response for each assay. E2 antibody titer: titer of immune serum generated to bind soluble E2Δ123 monomer. Homologous neutralization titer: ID_50_ titer that neutralizes gt‐1a HCVpp. Cross‐neutralization titer: the ID_50_ titer to neutralize gt‐3a and ‐5a HCVcc. CD4^+^ and CD8^+^ T‐cell response: frequency of gt‐1a IFNγ^+^ CD4^+^ or CD8^+^ T cells as a percentage of total CD4^+^ or CD8^+^ T‐cell (CD3^+^) frequencies. T‐cell cross‐reactivity: the frequency of all non‐gt‐1a (i.e., gt‐1b and/or ‐3a) IFNγ^+^ CD4^+^ or CD8^+^ T cells as a percentage of total CD4^+^ or CD8^+^ T‐cell (CD3^+^) frequencies. The scale represents a low median response (pink) to high median response (red), with numbering to the right of the chart indicating the highest and lowest values for each analysis. Gray represents analysis that was not performed for that regimen. LOD, limit of detection

For ease of reference, a comparison of each vaccine regimen tested, alongside HCV‐specific T‐cell and Ab responses, is shown in a heatmap (Figure 4).

## DISCUSSION

HCV vaccine strategies have so far used viral vectors or proteins to generate T‐cell or nAb responses, respectively. However, bivalent viral vectored vaccines encoding multiple conserved‐sequence Ags may generate pan‐genotypic T cells and nAbs, reduce the number of vaccines required, and use established Good Manufacturing Practice facilities to reduce costs and enhance the scalability of vaccine production for human trials.

As a prerequisite to developing a bivalent viral vector vaccine, we show that a ChAdOx1 vector encoding E2Δ123 generated a greater Ab titer after a single vaccination and enhanced CD81 binding‐inhibitory and nAb titers when combined with E2Δ123_HMW_ protein vaccine boosts compared to a homologous protein prime‐boost regimen, consistent with previous reports.^[^ ^29^ ^]^ Chmielewska et al. reported that both an Ad6E2_662_ and Ad6E1E2p7 prime plus two E1E2 protein boosts induced only modest nAbs in mice and did not increase nAb titers compared to three subsequent protein vaccinations in guinea pigs.^[^ ^30^ ^]^ Consistent with Chmielewska et al. and other reports of varying nAb titers using different E1/E2 forms,^[^ ^31^, ^32^ ^]^ our result highlights the importance of generating nAbs using an optimized E2 construct.

A ChAd‐E2Δ123 prime also broadened the Ab isotype repertoire compared to protein vaccines. T follicular helper cells (Tfh) play a critical role in GC‐mediated response, and the reciprocal IgG1/IgG2a titer may be used as a surrogate marker to measure levels of GC‐mediated Ab class switching. This was significantly lower for ChAd‐E2Δ123 immune sera than E2Δ123_HMW_ immune sera, indicating greater levels of ChAd‐induced class switching and GC activity. Generating broad Ab isotype repertoires using viral vectors was also reported using Ad6 in HCV^[^ ^30^ ^]^ and ChAd63 and MVA in malaria.^[^ ^33^ ^]^ It is expected that increased GC activity generates higher‐affinity Abs through somatic hypermutation, which may enhance Ab neutralizing activity against viral infection. Although we did not directly enumerate Tfh, our data suggest that a ChAd‐E2Δ123 prime alters GC‐mediated Ab response. Assessment of the bivalent vector vaccine further revealed that CD4^+^ T‐cell frequencies positively correlated with nAb titers, suggesting that an HCV vaccine that generates CD4^+^ T cells may enhance nAbs production.

Next, we show that ChAd and MVA vectors encoding the bivalent immunogen, Gt1‐6‐E2Δ123, used as the prime/boost, elicited high‐titer Abs, comparable to a monovalent ChAd‐E2Δ123 prime, MVA‐E2Δ123 boost regimen, with no difference detected in CD81‐inhibitory and HCVpp‐neutralizing titers. Furthemore, the prime/boost bivalent regimen generated a comparable gt‐1a‐specific and superior gt‐1b‐specific anti‐E2 CD4^+^ T‐cell response compared to the prime/boost monovalent vaccines, likely attributable to T‐cell targeting of pan‐gt E2 epitopes in the Gt1‐6 immunogen, with additional CD4^+^ and CD8^+^ T cells targeting conserved NS3‐5 epitopes. The monovalent E2Δ123 vaccines generated a greater gt‐1a‐specific anti‐E2 CD8^+^ T‐cell response than the bivalent E2Δ123‐Gt1‐6 vaccines, possibly explained by the absence of immunodominant NS3‐5 CD8 epitopes in the monovalent E2Δ123 vaccine enabling subdominant E2 epitopes to induce CD8^+^ T cells.

When comparing the prime/boost bivalent vaccine regimen with the prime/boost monovalent T‐cell vaccine regimen (Gt1‐6 encoded only), the bivalent vaccine regimen generated higher CD8^+^ and CD4^+^ T‐cell frequencies targeting gt‐1a, ‐1b, and ‐3a epitopes. Surprisingly, bivalent vaccines increased NS3‐5‐specific T‐cell responses compared to monovalent vaccines despite both immunogens containing the same Gt1‐6 epitopes—possible explanations for this include: (1) the potential additive effect of generating of E2Δ123‐specific T/B cells, which may have a bystander effect on NS‐specific T/B cells, a mechanism recently observed for the p30 T‐cell helper epitope,^[^ ^34^ ^]^ or (2) the furin 2A sequence (encoded between the Gt1‐6 and E2Δ123 sequences in the ChAd vector to generate two cleaved polypeptides during translation) is not 100% effective and therefore any noncleaved polypeptide may affect Ag stability, proteasomal processing, major histocompatibility complex cross‐presentation, and increase the overall immunogenicity compared to monovalent vaccines. Overall, consistent with a reported bivalent flu vaccine,^[^ ^35^ ^]^ the prime/boost bivalent HCV vaccine regimen generated robust Ab and T‐cell responses comparable to immune responses induced by both the monovalent Gt1‐6 and E2Δ123 prime/boost vaccine regimens.

To assess the effect of a third vaccination, we administered the bivalent immunogen as a ChAd‐Gt1‐6‐E2Δ123 prime, MVA‐Gt1‐6‐E2Δ123 boost (C/M), with an additional MVA‐Gt1‐6‐E2Δ123 boost (C/M/M), E2Δ123_HMW_ protein vaccine boost (C/M/P), or no additional boost (C/M/‐). These regimens were also compared against three protein immunizations (P/P/P), a regimen historically used to elicit high‐titer nAbs. We demonstrate that a ChAd‐Gt1‐6‐E2Δ123 prime, MVA‐Gt1‐6‐E2Δ123 boost (C/M) generates significantly higher nAb and Ab titers to E2Δ123 compared to three E2Δ123_HMW_ protein immunizations. An additional MVA‐Gt1‐6‐E2Δ123 or E2Δ123_HMW_ protein third vaccination did not increase Ab titers, contrary to some studies,^[^ ^33^, ^36^ ^]^ but consistent with others.^[^ ^37^, ^38^ ^]^ Previous studies using three or four immunizations have spaced vaccinations three weeks apart.^[^ ^26^, ^39^ ^]^ However, in this study, the C/M/M, C/M/P, and P/P/P vaccinations were deliberately spaced further apart to allow B‐cell maturation and somatic hypermutation to increase Ab affinity and nAb activity. Despite achieving higher Ab titers, nAb activity declined at week 22, suggesting an increase in nonfunctional Ab specificities. This suggests that the timing of booster vaccines must be carefully evaluated in future immunization regimens using this approach.

As expected, regimens using viral vector vaccines generated HCV‐specific CD4^+^ and CD8^+^ T‐cell responses that were significantly higher than the protein‐only regimen. A ChAd‐Gt1‐6‐E2Δ123 prime, MVA‐Gt1‐6‐E2Δ123 boost generated T‐cell responses that were sustained for up to 12 weeks post‐MVA. An additional bivalent MVA or E2Δ123_HMW_ protein third immunization did not further enhance the T‐cell response, in keeping with previous reports.^[^ ^37^, ^38^, ^40^ ^]^ Given that the bivalent HCV vaccine contains subdominant T‐cell epitopes within the Gt1‐6 immunogen,^[^ ^24^ ^]^ it is possible that these epitopes may be readily boosted by other vaccines modalities, such as virus‐like particles and nanoparticles. These approaches may be explored in future studies.

The lack of robust, immune‐competent small animal models to test HCV vaccine candidates remains a critical block in the field. The rat hepacivirus model is one model that we are currently exploring.^[^ ^41^, ^42^ ^]^ This model may be used to assess vaccine strategies using an HCV‐analogous viral challenge. However, ultimately, in the absence of an immune‐competent small animal model that supports robust HCV replication and recapitulates the pathogenesis of HCV, vaccine efficacy will be dependent on phase II human efficacy studies or the development of human challenge models.

In summary, a bivalent viral vector vaccine encoding two sequence‐optimized immunogens (E2Δ123 and conserved segment Gt1‐6 sequence) may be an optimal regimen to generate a balanced polyfunctional T‐cell and high‐titer nAb response. Developing vaccination strategies that combine two immunogens to generate T‐cell and nAb responses from a single construct has broader importance for the development of vaccines against other pathogens. Future studies assessing mRNA to deliver pan‐genotypic T‐ and B‐cell immunogens could be used as alternative, or in addition to, prime/boost stategies with viral‐vectored vaccines. Several studies have demonstrated that one of the best‐performing COVID‐19 vaccine regimens is ChAdOx1 prime followed by an mRNA boost with high spike‐specific broadly nAb, IgG, and IgA titers alongside CD4^+^ and CD8^+^ T cells.^[^ ^43^, ^44^, ^45^ ^]^ The use of ChAdOx1‐delivered Gt1‐6‐E2Δ123 followed by an mRNA Gt1‐6‐E2Δ123 boost may prove to be an optimal regimen for the generation of HCV‐specific T‐ and B‐cell responses.

## CONFLICT OF INTEREST

T.D., S.C., and E.B. are all contributors or inventors on patent PCT/GB2017/050840 that describes the conserved segment HCV Gt1‐6 T‐cell vaccine used in this study. H.D. and P.P. are named inventors on patents PCT/AU2007/001221 and PCT/AU2011/001534 that describe E2 antigens used in this study. H.D., E.B., and S.C. are named inventors of PCT/AU2021/050437.

## AUTHOR CONTRIBUTIONS

Conceptualization: Timothy Donnison, Joey McGregor, Senthil Chinnakannan, Rob J. Center, Pantelis Poumbourios, Paul Klenerman, Heidi E. Drummer, and Eleanor Barnes. Data curation: Timothy Donnison and Joey McGregor. Formal analysis: Timothy Donnison, Joey McGregor, Heidi E. Drumme, and Eleanor Barnes Supervision: Rob J. Center, Senthil Chinnakannan, Pantelis Poumbourios, Heidi E. Drummer, and Eleanor Barnes. Investigation: Timothy Donnison and Joey McGregor Methodology: Timothy Donnison, Joey McGregor, Senthil Chinnakannan, Claire Hutchings, Rob J. Center, Pantelis Poumbourios, Paul Klenerman, Heidi E. Drummer, and Eleanor Barnesr Project administration; Timothy Donnison, Rob J. Center, Heidi E. Drummer, and Eleanor Barnes. Resources: Pantelis Poumbourios, Paul Klenerman, Heidi E. Drummer, and Eleanor Barnes. Funding acquisition: Timothy Donnison, Rob J. Center, Pantelis Poumbourios, Heidi E. Drummer, and Eleanor Barnes. Visualization: Timothy Donnison and Joey McGregor, Writing – original draft: Timothy Donnison. Writing – review and editing: Timothy Donnison, Joey McGregor. Senthil Chinnakannan, Rob J. Center, Claire Hutchings, Pantelis Poumbourios, Paul Klenerman, Heidi E. Drummer, and Eleanor Barnes.

## Supporting information

Supplementary Material
