# Supplementary material for: A pan‐genotype hepatitis C virus viral vector vaccine generates T cells and neutralizing antibodies in mice
Source: Hepatology. 2022 May 19;76(4):1190–202. doi: 10.1002/hep.32470 (PMC9790311; doi:10.1002/hep.32470)
Supplement: Supplementary file 1 — Supplementary Material [file HEP-76-1190-s001.docx]

**SUPPLEMENTARY MATERIAL**

**SUPPLEMENTARY RESULTS**

**A ChAd-Gt1-6 and E2Δ123_HMW_ protein sequential vaccination generates both HCV-specific Ab and T-cells**

In order to assess the T-cell and Ab response from a single vaccine, we first demonstrate that a ChAd-Gt1-6 T-cell vaccine (design in **supplementary figure 1A**) and the E2Δ123_HMW_ protein vaccine mixed with Addavax^®^ (design in **supplementary figure 2A**) can be sequentially administered to elicit Ab and T-cell responses that are equivalent to each vaccine given alone (**supplementary figure 3A-F**). Animals vaccinated with ChAd-Gt1-6 had similar T-cell responses when given with or without a E2Δ123_HMW_ protein vaccine 2 days later (**supplementary figure 3F** group-6 v group-5). Ab responses were similar in animals that received E2Δ123_HMW_ protein vaccine regardless of whether a day-0 ChAd-Gt1-6 prime was administered (**supplementary figure 3B,** group-1 and -3 v group-4). Sequential administration of ChAd-Gt1-6 followed by E2Δ123_HMW_ protein vaccine generated immune sera targeting neutralization epitopes and with increased capacity for CD81-E2 inhibition compared to the sequential administration of ChAd-Gt1-6 with E2RBD_HMW_ protein vaccine boost (**supplementary figure 3C-D**). Therefore, we used the E2Δ123_HMW_ protein vaccine in subsequent regimens. Since only 2 animals across all regimens generated HCVpp nAbs (**supplementary figure 3E**), we elected to use longer intervals between vaccines in subsequent experiments to allow for the development of nAbs.

**SUPPLEMENTARY FIGURES**

**
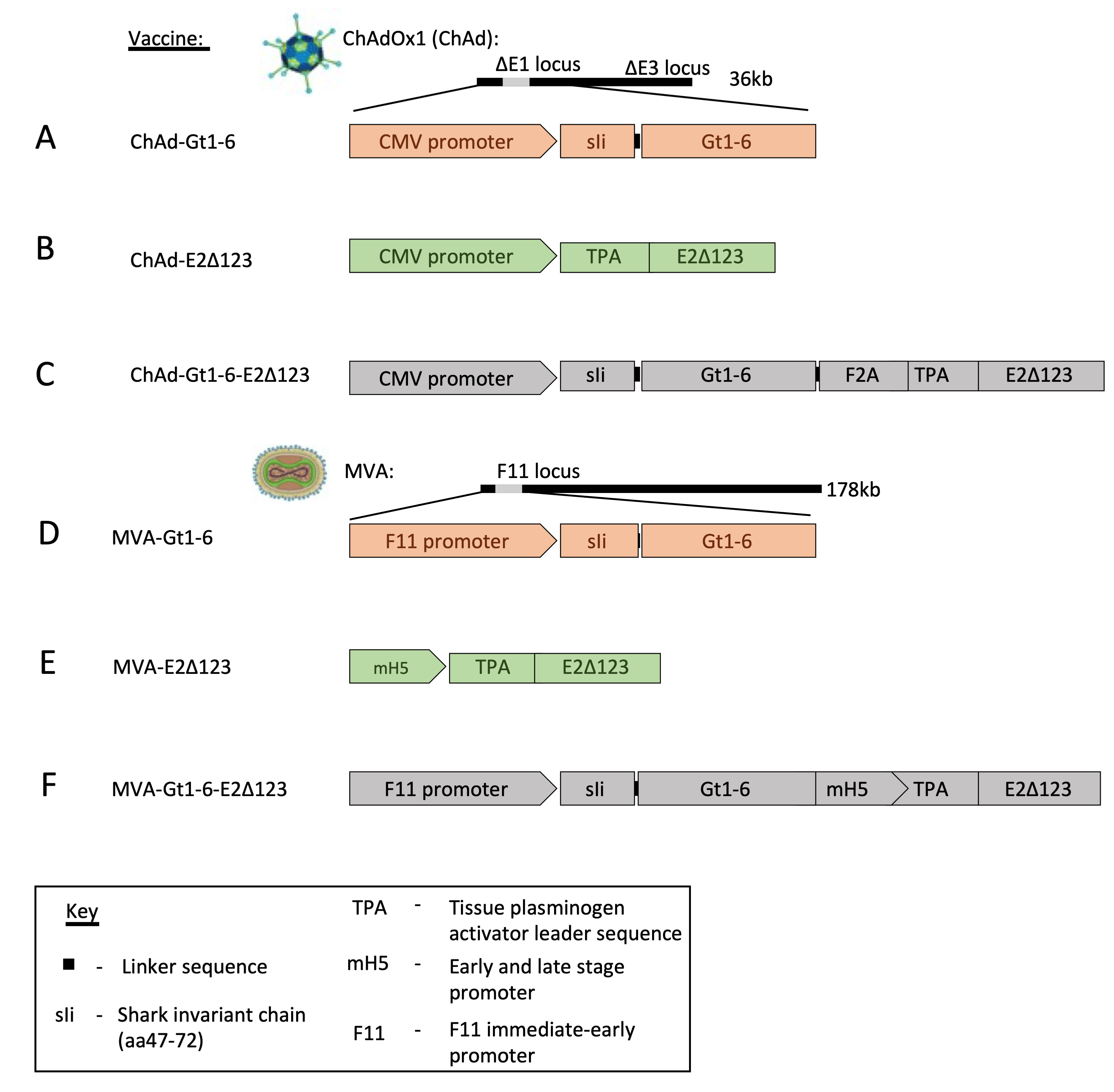
**

**Supplementary figure 1: HCV viral vector vaccine design.** (**A-C**) Schematic of ChAdOx1 vector vaccines. All transgenes were inserted in the E1 adenovirus locus (**A**: Gt1-6; **B**: E2Δ123; **C**: Gt1-6-E2Δ123). Each immunogen has a Kozak sequence and genetic adjuvant (either sIi;aa 47-72 or tissue plasminogen activator leader sequence [TPA]) encoded at the 5’ end. All ChAdOx1 vaccines contain a 5’ CMV promoter. Due to the limited sequence capacity of the ChAdOx1 vector, the bivalent immunogen (**C**) contains a furin 2A (F2A) cleavage sequence between the Gt1-6 and E2Δ123 sequences (instead of E2Δ123 having its own promoter) that causes the ribosome to skip over the F2A sequence in the mRNA transcript. (**D-F**) Schematic of modified vaccinia Ankara (MVA) vector vaccines. All transgenes are encoded in the F11 MVA locus (**D**: Gt1-6; **E**: E2Δ123; **F**: Gt1-6-E2Δ123). As with ChAdOx1 vectors, each immunogen has a Kozak sequence and genetic adjuvant (either sIi;aa 47-72 or tissue plasminogen activator leader sequence [TPA]) encoded at the 5’ end. MVA-Gt1-6 (**D**) uses an F11 promoter whereas the bivalent MVA vaccine (**F**) includes both an F11 and mH5 promoter (instead of an F2A sequence) for the Gt1-6 and E2Δ123 Ags, respectively. To ensure an accurate comparison between vaccines, the MVA-E2Δ123 (**E**) vaccine encodes the mH5 promoter instead of the F11 promoter to match the promoter used in the bivalent construct.


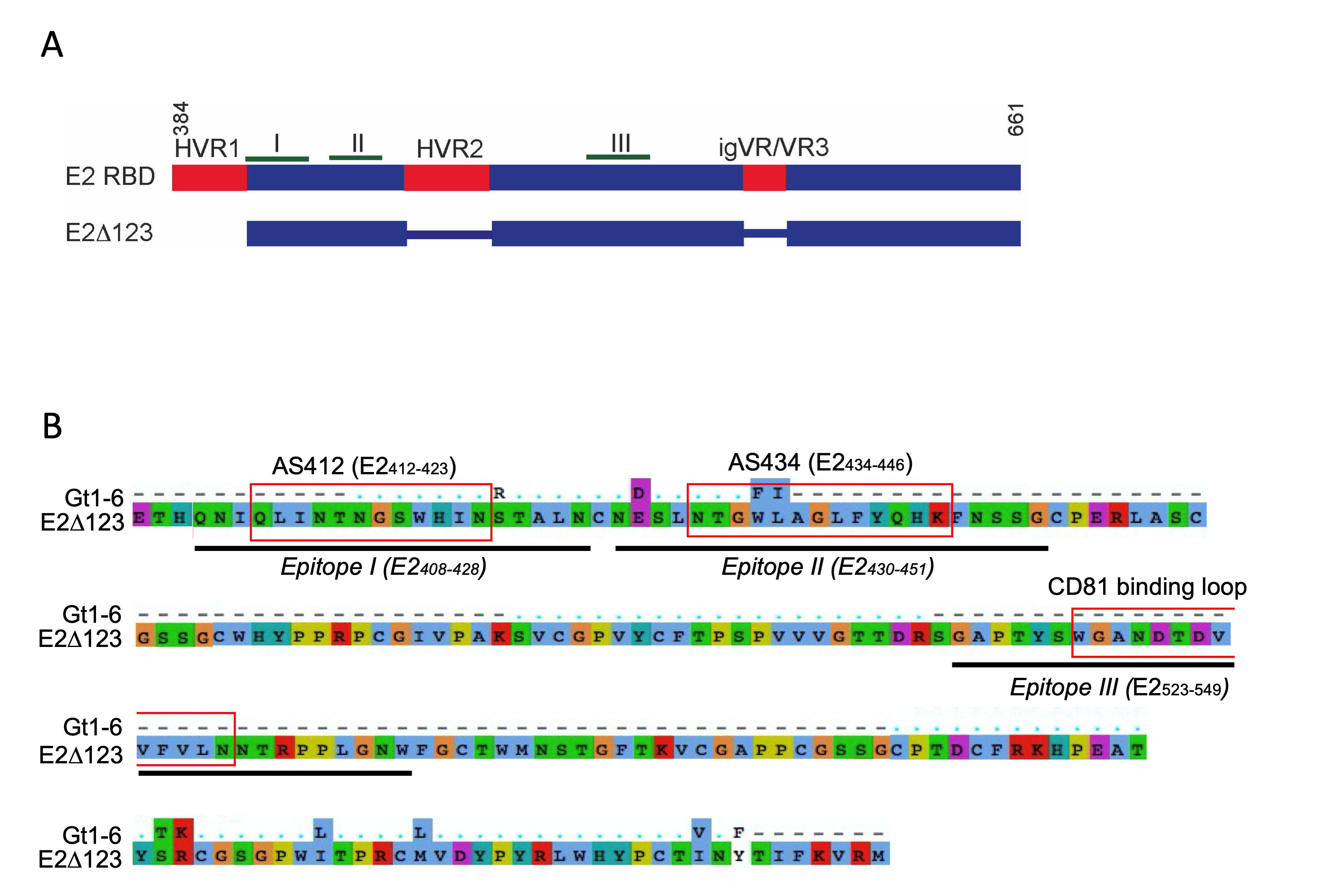


**Supplementary figure 2:** **E2 vaccine sequences** (**A**) Schematic representation (adapted from Alhammad et al., JVI. 2015) of E2RBD and E2Δ123 with replacement of truncated HVR1, HVR2, and igVR (red) with a GSSG linker in E2Δ123. Numbering is according to the H77c prototype strain. Epitope I, II, and III regions are underlined on the E2 structure and overlap CD81 binding sites: AS412, AS434, and CD81 binding loop, respectively. (**B**) E2 sequence comparison between Gt1-6 T-cell and E2Δ123 Ags. The E2Δ123 sequence is used as a reference sequence while black dashes in the Gt1-6 sequence indicate amino acids not present. Blue dots indicate amino acids shared with the E2Δ123 reference sequence. Epitope I, II, and III regions are underlined while the red boxes indicate CD81 binding sites: AS412, AS434, and CD81 binding loop.


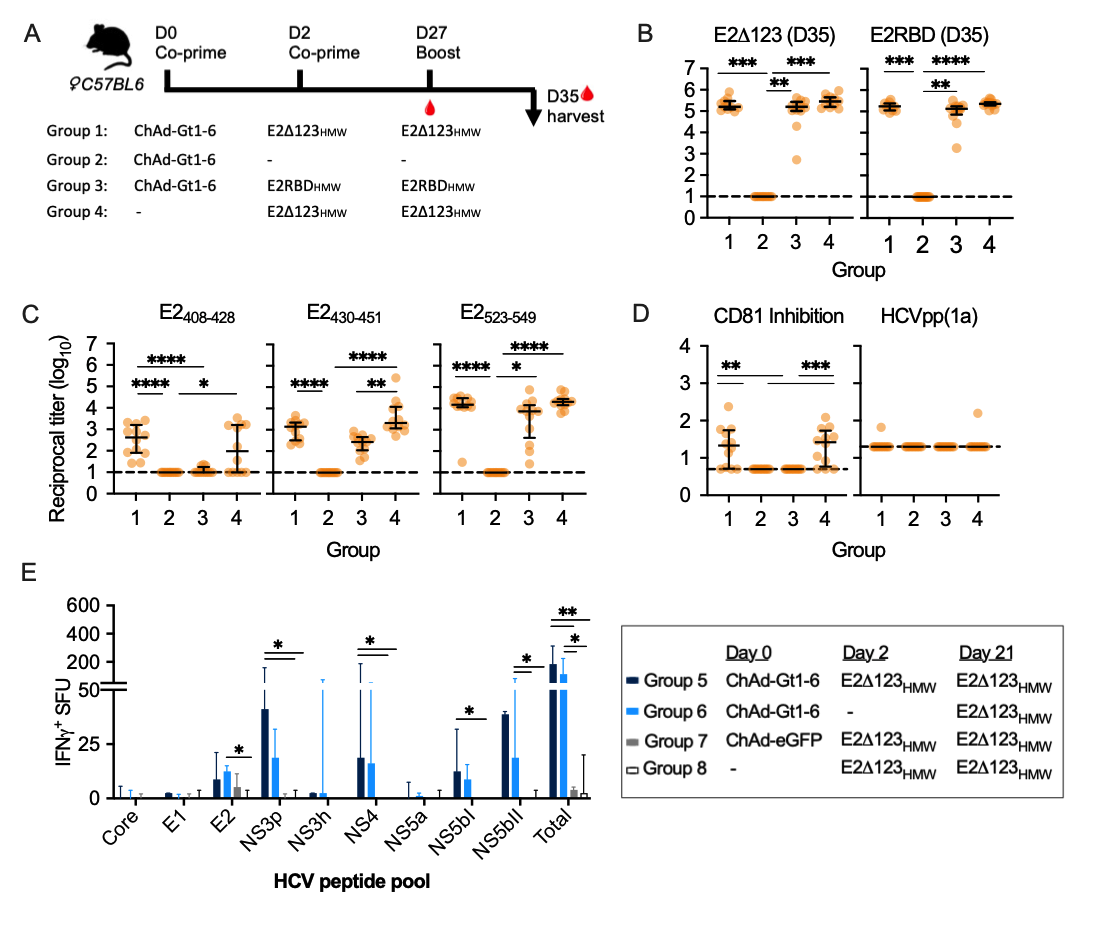


**Supplementary figure 3**: **Generation of T-cell and E2-specific Ab responses by** **sequential-administration of ChAd-Gt1-6 viral vector vaccine and E2Δ123_HMW_ protein vaccine.** (**A**) Groups of age-matched female *C57BL/6* mice were vaccinated with a sequential priming vaccine at day-0 and day-2 followed by a vaccination at week-4 (n=12/group). ChAd-Gt1-6 was given intramuscularly in the left quadricep at 10^8^ infectious units (IU) in 40μL sterile PBS and E2 proteins (20μg) were mixed 1:1 with 50μL of Addavax^®^ adjuvant and administered subcutaneously in the back of the neck. Both E2Δ123_HMW_ (E2 with HVR1, HVR2, and igVR regions deleted) and E2RBD_HMW_ (wildtype gt-1a E2 without variable regions deleted) were administered as their high molecular weight (HMW) species. (**B**) Day-35 ELISA assay to determine immune sera capacity to bind gt-1a E2Δ123 and E2RBD monomers. (**C**) Day-35 immune sera capacity to bind CD81-binding determinants: AS412 (E2_408-428_), AS434 (E2_430-451_), and CD81 binding loop 3 (E2_523-549_). ELISA Ab titres are measured at ten times background (BSA) using dilution curves and are plotted as log reciprocal titres. (**D**) Day-35 immune sera titres that inhibit 50% (ID50) of E2 binding to CD81 and HCVpp (gt-1a) entry into Huh7.5 cells (dashed line is the cut off for detectable responses). (**F**) A shortened 4-week regimen was used to assess the T-cell response. Splenocytes were harvested at day-28 post-prime vaccination and stimulated *ex vivo* using HCV peptides (15mer overlapping by 11aa) covering the length of the HCV proteome for gt-1a (H77). IFNγ^+^ spot forming units (SFU) were measured by ELISpot assay and plotted per 10^6^ splenocytes. The breadth of the day-28 vaccine-induced IFNγ^+^ ELISpot responses (plotted as IFNγ^+^ spot forming units (SFU) per 10^6^ splenocytes) are shown across gt-1a peptide pools corresponding to: core, E1, E2, NS3p, NS3h, NS4, NS5a, NS5bI (aa 2421-2718), NS5bII (aa 2719-2011), and total response (total defined as the accumulative SFU of individual positive peptide pools where positive is defined as greater than the DMSO negative control plus three standard deviations). All bars are medians and interquartile ranges are displayed. The D’Agostino and Pearson test was used to determine normality of data distribution and Kruskal-Wallis with multiple comparisons were performed to determine significant differences between two group medians at a 95% confidence interval. *P* values indicate significant difference between groups when < 0.05*, <0.01**, <0.001***, <0.0001****.


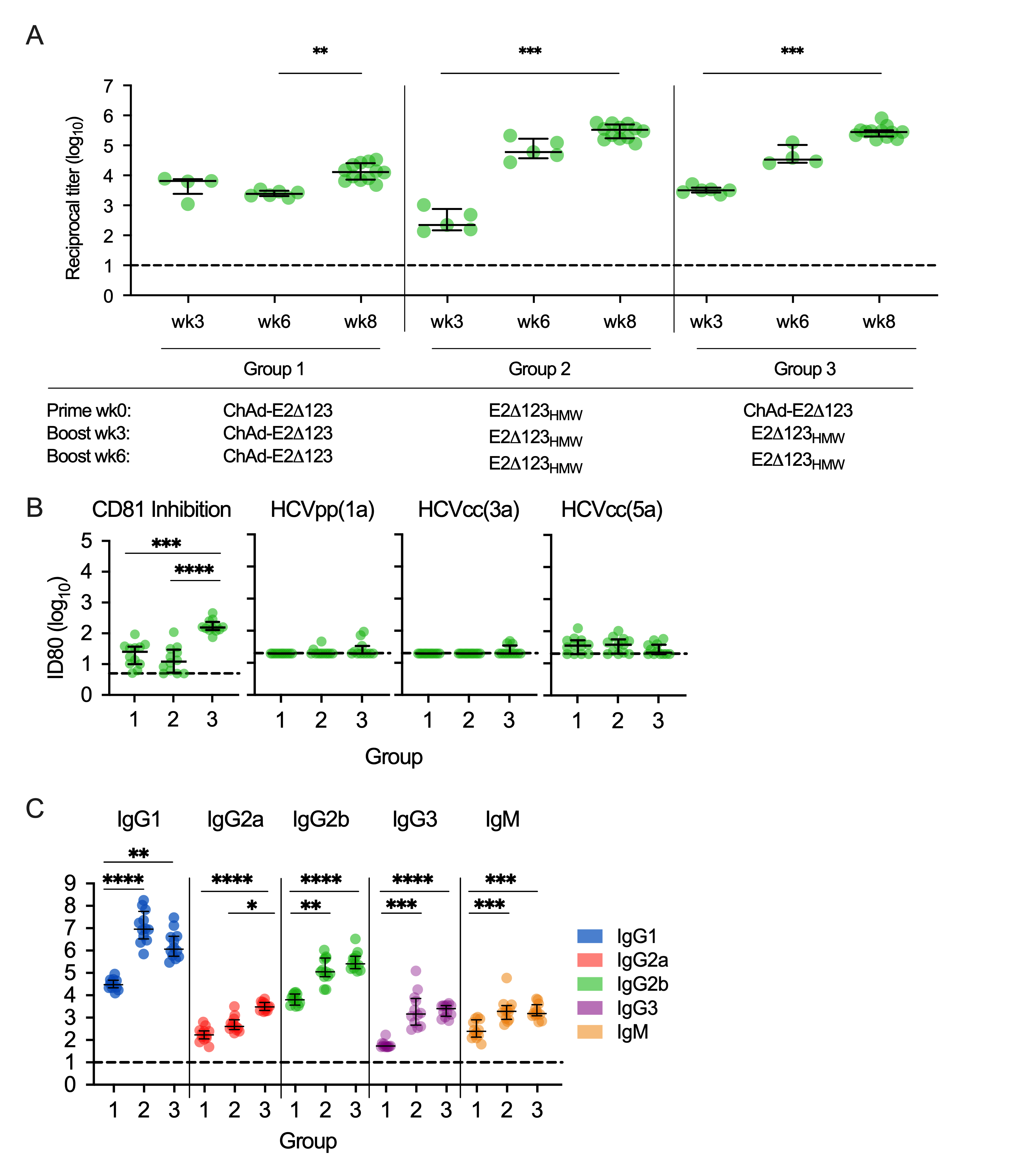


**Supplementary figure 4: Immunogenicity of an E2Δ123 protein sequence encoded in a ChAdOx1 prime vaccine**. Groups of twelve age-matched female *C57BL/6* mice were vaccinated with a prime vaccine at week-0, a boost vaccine at week-3 (W3), and another boost vaccine at week-6 (W6), followed by a terminal bleed at week-8 (W8). Vaccination groups are described in detail in figure 1. (**A**) Comparison of E2-specific Ab titres at week-3 (pre-boost 1), week-6 (pre-boost 2), and at week-8 (end of study, EOS) determined by ELISA assay. (**B**) EOS immune sera that inhibit 80% (ID80) of: E2 binding to CD81, HCVpp (gt-1a) and HCVcc (gt-3a and -5a) virion entry into cells (dashed line is cut off for detectable responses). ID80 inhibitory titres were determined using a dilution curve where the vaccine-induced Ab response was background subtracted and standardised to a negative control which displayed 100% binding/entry (e.g., BSA, instead of immune sera, incubated with E2 or HCVpp/cc prior to determining CD81 binding or cell infectivity). (**C**) Week-8 isotype-specific Ab titres. All bars are medians and interquartile ranges are displayed. The D’Agostino and Pearson test was used to determine normality of data distribution and Kruskal-Wallis with multiple comparisons were performed to determine significant differences between two group medians at a 95% confidence interval. *P* values indicate significant difference between groups when < 0.05*, <0.01**, <0.001***, <0.0001****.


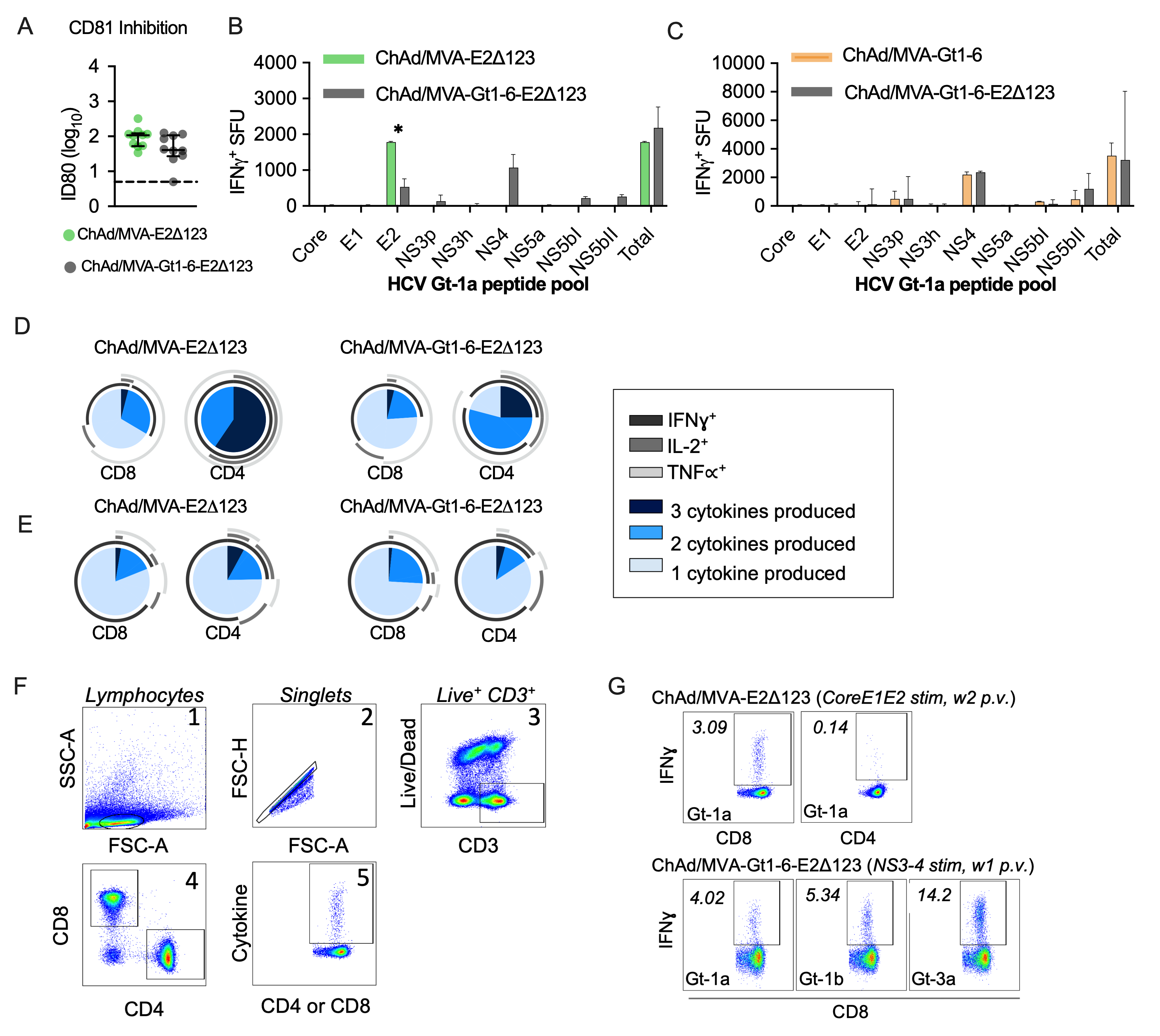


**Supplementary figure 5: Immunogenicity of a bivalent HCV vaccine immunogen—Gt1-6-E2Δ123—compared to monovalent immunogens, Gt1-6 and E2Δ123, in a ChAdOx1 prime, MVA boost regimen.** Experiment groups and regimens provided in figure 2. (**A**) Immune sera were assessed for reactivity to capacity to inhibit 80% binding of CD81 to E2 binding. ID80 inhibitory titres were determined using a dilution curve where the vaccine-induced Ab response was background subtracted and standardised to a negative control which displayed 100% binding/entry (e.g., BSA, instead of immune sera, incubated with E2 prior to determining CD81 binding or cell infectivity). Dashed line is the cut off for detectable responses). (**B-C**) Assessment of week-6 (group-1 and -2; **B**) and week-5 (group-3 and -4; **C**) vaccine-induced T-cell responses via *ex vivo* IFNγ ELISpot assay using HCV peptides (15mer overlapping by 11aa) covering the length of the HCV proteome for gt-1a (H77). (**D-E**) Week-6 (group-1 and -2; **D**) and week-5 (group-3 and -4; **E**) vaccine-induced T-cell polyfunctionality was determined by ICS and flow cytometry after splenocyte stimulation *ex vivo* using HCV peptides (15mer overlapping by 11aa) covering the length of the HCV proteome for gt-1a (H77) to detect produced cytokines, IFNγ, TNFα, and IL-2. Pie charts are medians and calculated using Pestle and SPICE software. (**F**) The intracellular cytokine stain (ICS) gating strategy is shown. Briefly, lymphocytes were gated first, then singlets, then live CD3^+^ lymphocytes (T-cells), then either CD4^+^ or CD8^+^ T-cells, then cytokine positive cells. (**G**) Example ICS plots are shown. All data are plotted as medians and interquartile ranges. The D’Agostino and Pearson test was used to determine normality of data distribution and Mann-Whitney were performed to determine significant differences between two group medians at a 95% confidence interval. *P* values indicate significant difference between groups when < 0.05*, <0.01**, <0.001***, <0.0001****.


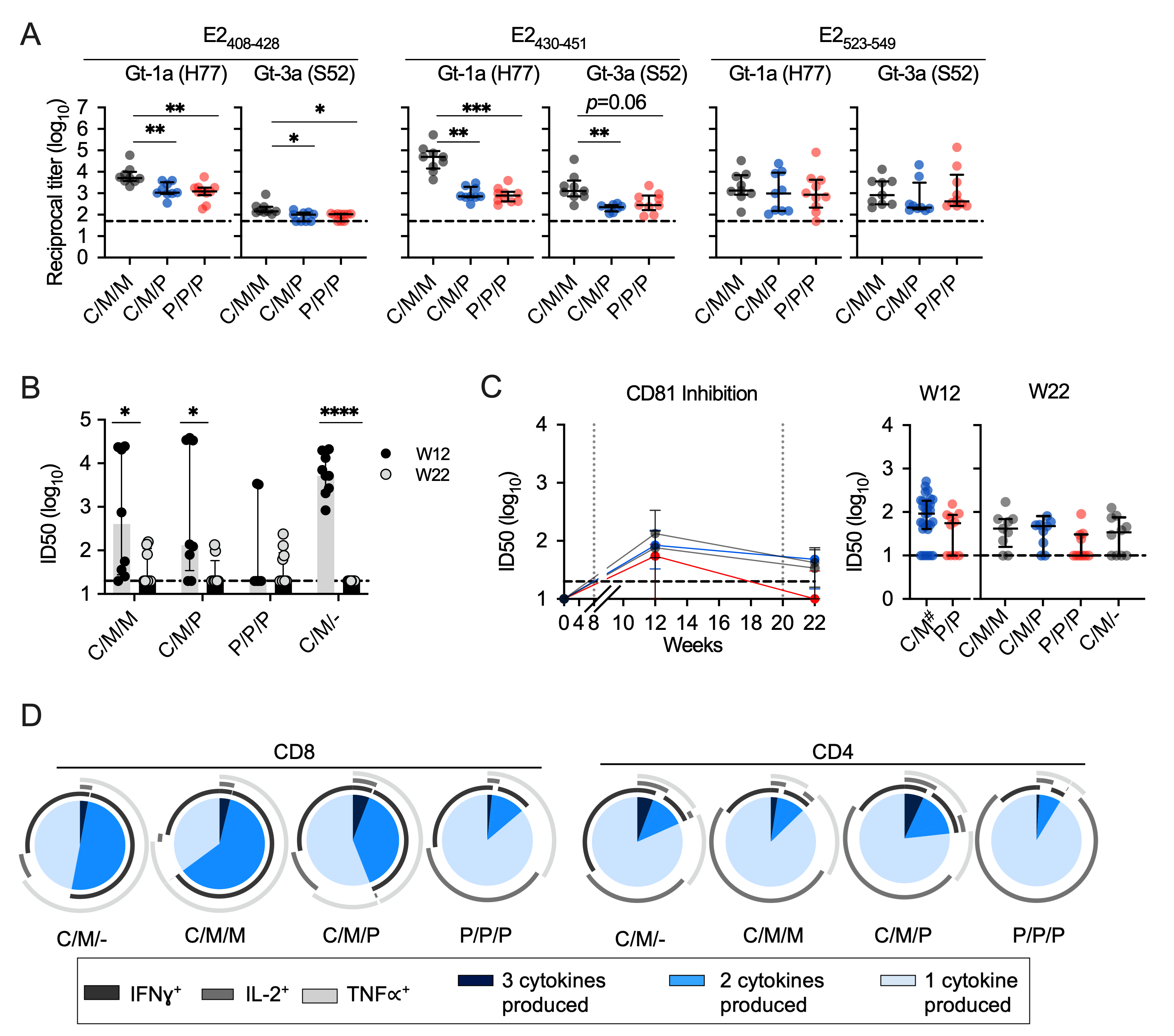


**Supplementary figure 6: Assessment of mixed modality vaccination regimens using bivalent HCV vaccines E2Δ123_HMW_ protein vaccines**. Groups of ten age-matched female *C57BL/6* mice were vaccinated with a prime vaccine at day-0, a boost vaccine at week-8 (W8), and another boost vaccine at week-20 (W20), followed by a terminal bleed at week-22 (W22), as described in figure 3. (**A**) Immune sera’s capacity to bind E2Δ123 and both gt-1a (H77) and gt-3a (S52) sequence variants of AS412 (E2_408-428_), AS434 (E2_430-451_), and the CD81 binding loop (E2_523-549_). (**B**) Comparison of group-specific HCVpp(gt-1a) ID50 titres between week-12 and -22 (dashed line is the cut off for detectable responses). (**C**) Longitudinal CD81 inhibition assay analysis of immune sera collected at week-12 and -22 and plotted as ID50 (log10) (Dashed line is the cut off for detectable responses). (**D**) Assessment of week-22 vaccine-induced T-cell polyfunctionality via assessment of secreted cytokines, IFNγ, TNFα, and IL-2 in ICS and flow cytometry, and calculated using Pestle and SPICE software. Pie charts are medians. All data are plotted as medians and interquartile ranges. The D’Agostino and Pearson test was used to determine normality of data distribution and Mann-Whitney were performed to determine significant differences between two group medians at a 95% confidence interval (Kruskal-Wallis test for multiple groups). *P* values indicate significant difference between groups when < 0.05*, <0.01**, <0.001***, <0.0001****.

**SUPPLEMENTARY TABLES**

**Supplementary Table 1**

| **Peptides** | | **Sequence** |
| --- | --- | --- |
| 408-428 | H77c | Biotin – KQNIQLINTNGSWHINSTALN-NH_2_ |
|  | S52 | Biotin – KQKLQLVNTNGSWHINSTALN-NH_2_ |
| 430-451 | H77c | Biotin - NESLNTGWLAGLFYQHKFNSSG-NH_2_ |
|  | S52 | Biotin - NESINTGFIAGLFYYHKFNSTG-NH_2_ |
| 523-549 | H77c | Biotin - GAPTYSWGANDTDVFVLNNTRPPLGNW-NH_2_ |
|  | S52 | Biotin - GRPTYNWGENETDVFLLESLRPPSGRW-NH_2_ |

**Supplementary Table 2**

| **Antibody** | **Produced / Catalogue number** | **Reference / Manufacturer** |
| --- | --- | --- |
| 2A12 | In house | (1) |
| mAb24 | In house | (2) |
| HC84.27 | In house | (3) |
| HCV1 | In house | (4) |
| mAb44 | In house | (2) |
| AR3C | In house | (5) |
| HC84.1 | In house | (3) |
| mAb25 | In house | (2) |
| mAb64 | In house | (2) |
| IFNγ-mAb (AN18) | 3321-3-250 | Mabtech |
| IFNγ- mAb R4-6A2 biotinylated | 3321-6-250 | Mabtech |
| Anti-biotin alkaline phosphatase | SA-5100-1 | Vector Laboratories |
| TNFα-FITC | 11-7321-82 | eBioscience |
| IFNγ-PE | 12-7311-82 | eBioscience |
| CD8-PerCP-Cy5.5 | 45-0081-82 | eBioscience |
| CD3-EF450 | 48-0032-82 | eBioscience |
| CD3-EF506 | 69-0032-82 | eBioscience |
| IL-2-APC | 503810 | Biolegend |
| CD4-AF700 | 100430 | Biolegend |
| CD4-BV605 | 100547 | Biolegend |
| Goat anti-IgG1 | ISO2-1KT | Sigma-Aldrich |
| Goat anti-IgG2a | ISO2-1KT | Sigma-Aldrich |
| Goat anti-IgG2b | ISO2-1KT | Sigma-Aldrich |
| Goat anti-IgG3 | ISO2-1KT | Sigma-Aldrich |
| Goat anti- human - HRP | P0214 | Agilent technologies (Dako) |
| Rabbit anti-mouse - HRP | P0260 | Agilent technologies (Dako) |
| Donkey anti-goat – HRP | PA1-28664 | ThermoFisher Scientific |

**REFERENCES**

1. Khan AG, Whidby J, Miller MT, Scarborough H, Zatorski AV, Cygan A, Price AA, Yost SA, Bohannon CD, Jacob J, Grakoui A, Marcotrigiano J. 2014. Structure of the core ectodomain of the hepatitis C virus envelope glycoprotein 2. Nature 509:381-4.

2. Alhammad Y, Gu J, Boo I, Harrison D, McCaffrey K, Vietheer PT, Edwards S, Quinn C, Coulibaly F, Poumbourios P, Drummer HE. 2015. Monoclonal Antibodies Directed toward the Hepatitis C Virus Glycoprotein E2 Detect Antigenic Differences Modulated by the N-Terminal Hypervariable Region 1 (HVR1), HVR2, and Intergenotypic Variable Region. J Virol 89:12245-61.

3. Krey T, Meola A, Keck ZY, Damier-Piolle L, Foung SK, Rey FA. 2013. Structural basis of HCV neutralization by human monoclonal antibodies resistant to viral neutralization escape. PLoS Pathog 9:e1003364.

4. Morin TJ, Broering TJ, Leav BA, Blair BM, Rowley KJ, Boucher EN, Wang Y, Cheslock PS, Knauber M, Olsen DB, Ludmerer SW, Szabo G, Finberg RW, Purcell RH, Lanford RE, Ambrosino DM, Molrine DC, Babcock GJ. 2012. Human monoclonal antibody HCV1 effectively prevents and treats HCV infection in chimpanzees. PLoS Pathog 8:e1002895.

5. Kong L, Giang E, Nieusma T, Kadam RU, Cogburn KE, Hua Y, Dai X, Stanfield RL, Burton DR, Ward AB, Wilson IA, Law M. 2013. Hepatitis C virus E2 envelope glycoprotein core structure. Science 342:1090-4.
